# MR-guided cardiac interventions using MR-compatible devices: first- in -man clinical trial

**DOI:** 10.1186/1532-429X-13-S1-O55

**Published:** 2011-02-02

**Authors:** Aphrodite Tzifa, Shakeel Qureshi, Gabriele Krombach, Aaron Bell, Gerald Greil, Philipp Beerbaum, Tobias Schaeffter, Thomas Krasemann, Eric Rosenthal, Reza Razavi

**Affiliations:** 1 King's College London, London, UK; 2 Aachen University Hospital, Aachen, Germany; 3 Guy's & St Thomas' Hospital, London, UK

## Introduction

Percutaneous cardiac interventions are currently performed under X-ray guidance. MR-guided cardiac interventions have been previously described but mainly in animals. Their translation into humans has been limited by the lack of MR-compatible and safe equipment, such as MR guidewires with mechanical characteristics similar to standard guidewires.

## Purpose

We are presenting the initial results of the first-in-man clinical trial on MR-guided interventions in patients with congenital heart disease.

## Methods

Ethics and UK regulatory authority approval were obtained prior to commencement of the trial. Inclusion criteria included patients above 2 years of age with great arterial valve stenosis, branch pulmonary artery or aortic arch stenosis and patients that needed balloon dilation of existing stents.

## Results

Seven patients aged 3 - 64 years have been recruited so far. Five patients have undergone successful interventions for pulmonary valve stenosis (n=4) and native aortic coarctation (n=1). One patient with left pulmonary artery stent underwent right heart catheterisation with the aid of the new MR-wire, but the gradient across the stent was only 5mmHg, hence no intervention was required. The last patient (8 year old child) with severe aortic stenosis had an unsuccessful attempt at ballooning the aortic valve, due to the inability of turning the wedge catheter into the ascending aorta, as the balloon of the catheter kept being pushed back by the aortic stenosis jet. Catheter manipulations were monitored with real time MRI sequence with interactive modification of imaging plane and slice position. Temporal resolution was 11-12 frames/sec. Median procedure and catheterisation times were 180 and 110 min, respectively. All patients were discharged home the day after the procedure with > 50% reduction of the stenosis gradient and no procedural complications.

## Conclusions

Initial results of the first-in-man clinical trial on MR-guided cardiac interventions are encouraging that with the availability of the new MR compatible and safe guidewire, certain percutaneous cardiac interventions will become feasible to perform solely under MR-guidance in the future.

